# The coincidental association between isotretinoin therapy and onset of pachydermodactyly: A case report

**DOI:** 10.1016/j.jdcr.2026.05.032

**Published:** 2026-05-19

**Authors:** Jessi M. Larrinaga, Shalini Bajaj, Joseph V. Caravaglio

**Affiliations:** a Dermatology Southeast, Jacksonville, Florida; b Division of Pediatrics, University of Central Florida College of Medicine, Nemours Children's Hospital, Orlando, Florida; c Division of Dermatology, University of Central Florida College of Medicine, Orlando, Florida

**Keywords:** acne vulgaris, benign fibromatosis, case report, general dermatology, isotretinoin, medical dermatology, musculoskeletal, pachydermodactyly, pediatric rheumatology, pediatric dermatology, rheumatology

## Introduction

Isotretinoin is a cornerstone therapy for severe, recalcitrant acne but is associated with a wide spectrum of side effects, primarily involving the skin, mucous membranes, and musculoskeletal (MSK) system. Well-documented MSK side effects include arthralgia, myalgia, arthritis, back pain, and, less commonly, tendon and ligament abnormalities.^1^, ^2^, ^3^ Pachydermodactyly (PDD) is a rare, benign dermatologic condition characterized by localized thickening of the skin over the proximal and distal interphalangeal joints.^4^ Its etiology remains elusive, with proposed mechanisms including repetitive mechanical irritation, systemic inflammation, and genetic predisposition.^4^^,^^5^ To date, no reports have linked isotretinoin therapy with PDD. Here, we describe the coincidental development of PDD in a 17-year-old male undergoing isotretinoin therapy for severe acne and discuss the clinical course and potential pathophysiologic mechanisms without implying causation.

## Case presentation

A 17-year-old male with autism spectrum disorder (ASD), nonverbal and dependent on typed communication, presented with severe, treatment-resistant acne involving the face, chest, and back. Prior oral antibiotics and topical therapies were ineffective. In March 2024, isotretinoin was initiated at 30 mg daily and titrated to 30 mg twice daily over 5 months.

**Fig 1:**
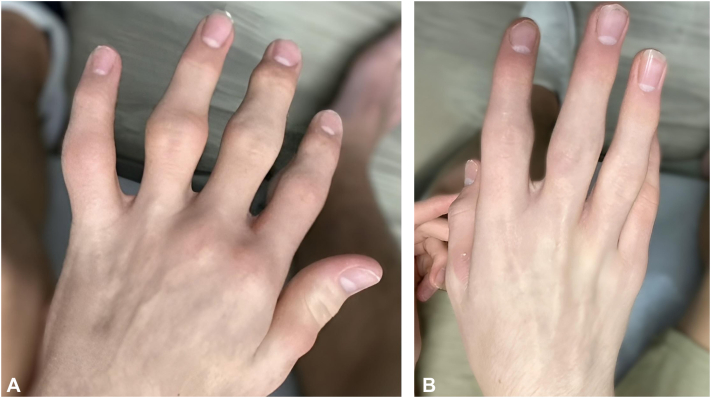
Initial presentation demonstrating bilateral swelling around the finger joints during the final month of isotretinoin therapy, **(A)** left hand and **(B)** right hand.

The patient showed marked improvement in acne, with significant reduction in active lesions by August 2024. In September 2024, during the final month of therapy, his family observed acute swelling of multiple bilateral proximal and distal interphalangeal (PIP/DIP) joints (Fig 1). The swelling was soft, mobile, fluctuant, and nontender, without warmth, erythema, systemic symptoms, or reported repetitive trauma. No new medications were introduced.

**Fig 2:**
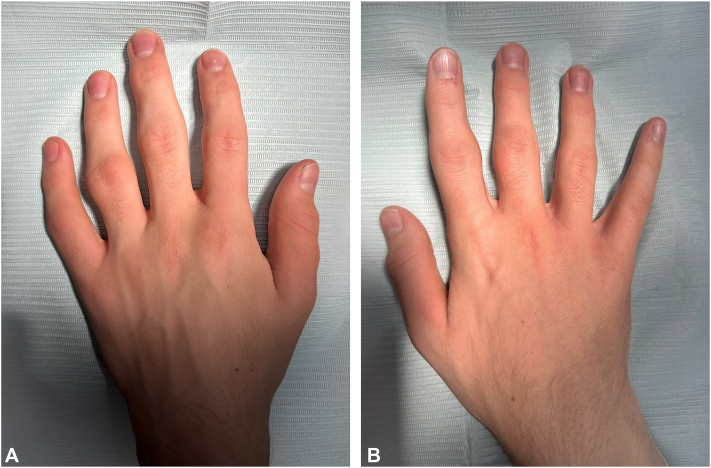
Follow-up images demonstrating persistent bilateral swelling around the finger joints several months after isotretinoin discontinuation and after initiation presentation, **(A)** left hand and **(B)** right hand.

Isotretinoin was discontinued, and prednisone (0.5 mg/kg/d) was initiated with taper. Pediatric rheumatology evaluation in October 2024 confirmed a clinical diagnosis of PDD, with unremarkable radiographic findings. The condition stabilized without progression, and by December 2024 there was no further swelling or systemic involvement. In March 2025, intralesional Kenalog was initiated for cosmetically bothersome areas (Fig 2). While the temporal association with isotretinoin is notable, causation cannot be established, highlighting the importance of recognizing rare conditions during acne treatment.

## Discussion

Isotretinoin, a synthetic retinoic acid derivative with functional similarities to Vitamin A, exerts its effects via nuclear retinoic acid receptors, influencing cellular differentiation, growth, and apoptosis.^6^ It is widely used as a treatment for severe acne vulgaris due to its effects on sebaceous activity, keratinization, and inflammation.^7^^,^^8^ In addition to dermatologic effects, isotretinoin is associated with musculoskeletal side effects including arthralgia, myalgia, tendinitis, and, rarely, tendon or ligament abnormalities.^1^, ^2^, ^3^ More severe skeletal effects, such as premature epiphyseal closure, bone density changes, and hyperostosis, have been reported, particularly with prolonged therapy.^9^^,^^10^ While mild MSK symptoms are common, severe skeletal manifestations typically occur with extended or high-dose exposure.^10^ Notably, associations with isotretinoin and specific conditions such as PDD have not been described.

PDD is a rare, benign condition characterized by progressive thickening of skin and soft tissue of the fingers, most commonly at the PIP and DIP joints. First described by Cushing and Bailey in 1931,^4^ it typically affects young males aged 10 to 30 years.^5^^,^^11^ It is usually painless, gradual in onset, and presents without systemic symptoms or abnormal inflammatory markers.^4^^,^^5^^,^^11^ Imaging demonstrates soft tissue swelling without bone abnormalities, while histopathology shows hyperkeratosis and thickened collagen fibers in the dermis.^12^^,^^13^

Although the etiopathogenesis of PDD remains unclear, it is believed to involve repetitive mechanical trauma, often associated with behaviors such as finger rubbing or interlacing. Associations with obsessive-compulsive disorder, anxiety, athletic activities, tuberous sclerosis, and Ehlers-Danlos syndrome have been reported.^5^^,^^11^ To date, no medication associations have been established. Isotretinoin may theoretically contribute to PDD in predisposed individuals. Its known effects on xerosis and retinoid dermatitis, particularly in friction-prone areas such as the hands, may increase mechanical irritation.^7^ These skin changes may lead to heightened mechanical irritation, including frequent rubbing or pressure on the fingers, which could contribute to the thickening of skin and soft tissues. Additionally, its influence on collagen synthesis and tissue remodeling may predispose the skin to dermal thickening under repetitive mechanical forces.^14^ While no causal relationship has been established, isotretinoin may exacerbate underlying vulnerabilities, facilitating the manifestation of PDD in susceptible individuals. Although this case does not suggest causality, it underscores that joint swelling during isotretinoin therapy should not be presumed to represent inflammatory arthropathy alone. In this context, it is essential to consider alternative explanations for such symptoms and to maintain a broad differential diagnosis.

The differential diagnosis of PDD in a 17-year-old patient includes isotretinoin-induced arthritis, juvenile idiopathic arthritis (JIA), hypertrophic osteoarthropathy (HOA), psoriatic arthritis, systemic sclerosis, and sarcoidosis. Isotretinoin-related arthritis typically presents with joint pain, whereas PDD presents as painless, spindle-shaped swelling of the fingers.^4^^,^^5^ JIA presents with joint swelling and inflammatory symptoms, such as a fever and rash, along with elevated markers.^15^ HOA is associated with clubbing and periostosis, often secondary to systemic disease such as underlying malignancy or chronic infections.^16^ Psoriatic arthritis presents with dactylitis, joint swelling, and personal or family history of psoriasis.^17^ Systemic sclerosis involves progressive fibrosis with potential internal organ involvement, and autoantibody positivity.^18^ Sarcoidosis, a granulomatous disorder, may present with MSK involvement and systemic features, with non-caseating granulomas on histology.^19^

Accurate differentiation of these conditions involves a thorough patient history, physical examination, laboratory tests, and imaging studies. Laboratory investigations typically include a complete blood count, erythrocyte sedimentation rate, C-reactive protein, rheumatoid factor, and antinuclear antibodies. Radiographic imaging, such as hand radiographs, can help assess for soft tissue swelling, bone abnormalities, or periostosis, which may point to specific diagnoses. In cases where PDD is suspected, a skin biopsy may be performed, revealing characteristic findings of hyperkeratosis and thick collagen fibers in the dermis and can aid in excluding alternative diagnoses.

In summary, while the etiology of PDD remains uncertain, this case suggests a potential, non-causal association with isotretinoin use in a genetically or mechanically predisposed individual. This report is presented not to establish a direct causal link, but to encourage further investigation into the possible MSK effects of isotretinoin. The patient’s clinical presentation and the temporal association with isotretinoin therapy underscore the importance of considering isotretinoin as a contributing factor in joint or soft tissue swelling, particularly in individuals predisposed by neurodevelopmental conditions like autism or by repetitive strain from constant device use.

## Conclusion

This case highlights a rare diagnosis of pachydermodactyly in a patient undergoing isotretinoin therapy for severe acne. It emphasizes the importance of a broad differential diagnosis, as not all joint swelling in this setting reflects inflammatory arthropathy. While isotretinoin is associated with MSK side effects, its cutaneous effects, such as xerosis and retinoid dermatitis, may contribute to mechanical irritation and potentially predispose susceptible individuals to conditions like PDD. This case serves to remind providers of the need for careful evaluation of MSK symptoms in patients receiving isotretinoin, particularly when considering rare diagnoses such as PDD.

## Conflicts of interest

Dr. Caravaglio has served as a speaker for Janssen Pharmaceuticals and AbbVie Pharmaceuticals. The remaining authors have no conflict of interest to declare.
